# The Association between Maternal Urinary Phthalate Concentrations and Blood Pressure in Pregnancy: A Systematic Review and Meta-Analysis

**DOI:** 10.3390/metabo13070812

**Published:** 2023-06-30

**Authors:** Mengyue Zhang, Jianchao Qiao, Pinpeng Xie, Zhuoyan Li, Chengyang Hu, Fei Li

**Affiliations:** 1 Department of Clinical Medicine, The Second School of Clinical Medicine, Anhui Medical University, 81 Meishan Road, Hefei 230032, China; 2114010147@stu.ahmu.edu.cn (M.Z.); 2013010481@stu.ahmu.edu.cn (J.Q.); 1913010662@stu.ahmu.edu.cn (P.X.); 2113010464@stu.ahmu.edu.cn (Z.L.); 2 Department of Prevention and Health Care, The First Affiliated Hospital of Anhui Medical University, Hefei 230022, China; 3 Department of Humanistic Medicine, School of Humanistic Medicine, Anhui Medical University, 81 Meishan Road, Hefei 230032, China; 4 Department of Epidemiology and Biostatistics, School of Public Health, Anhui Medical University, 81 Meishan Road, Hefei 230032, China

**Keywords:** phthalate, blood pressure, hypertensive disorders in pregnancy, gestational hypertension

## Abstract

Phthalates are commonly found in a wide range of environments and have been linked to several negative health outcomes. While earlier research indicated a potential connection between phthalate exposure and blood pressure (BP) during pregnancy, the results of these studies remain inconclusive. The objective of this meta-analysis was to elucidate the relationship between phthalate exposure and BP in pregnancy. A comprehensive literature search was carried out with PubMed, EMBASE, and Web of Science, and pertinent studies published up until 5 March 2023 were reviewed. Random-effects models were utilized to consolidate the findings of continuous outcomes, such as diastolic and systolic BP, as well as the binary outcomes of hypertensive disorders of pregnancy (HDP). The present study included a total of 10 studies. First-trimester MBP exposure exhibited a positive association with mean systolic and diastolic BP during both the second and third trimesters (β = 1.05, 95% CI: 0.27, 1.83, I^2^ = 93%; β = 0.40, 95% CI: 0.05, 0.74, I^2^ = 71%, respectively). Second-trimester monobenzyl phthalate (MBzP) exposure was positively associated with systolic and diastolic BP in the third trimester (β = 0.57, 95% CI: 0.01, 1.13, I^2^ = 0; β = 0.70, 95% CI: 0.27, 1.13, I^2^ = 0, respectively). Conversely, first-trimester mono-2-ethylhexyl phthalate (MEHP) exposure demonstrated a negative association with mean systolic and diastolic BP during the second and third trimesters (β = −0.32, 95% CI: −0.60, −0.05, I^2^ = 0; β = −0.32, 95% CI: −0.60, −0.05, I^2^ = 0, respectively). Additionally, monoethyl phthalate (MEP) exposure was found to be associated with an increased risk of HDP (OR = 1.12, 95% CI: 1.02, 1.23, I^2^ = 26%). Our study found that several phthalate metabolites were associated with increased systolic and diastolic BP, as well as the risk of HDP across pregnancies. Nevertheless, given the limited number of studies analyzed, additional research is essential to corroborate these findings and elucidate the molecular mechanisms linking phthalates to BP changes during pregnancy.

## 1. Introduction

Hypertensive disorders of pregnancy (HDP) rank among the top contributors to maternal and neonatal mortality and morbidity [1,2]. Hypertension can either be chronic or develop during pregnancy and may be accompanied by other comorbidities, potentially resulting in pre-eclampsia (PE) and preterm birth (PTB) [3]. Factors known to heighten the risk of elevated blood pressure (BP) during pregnancy include being overweight or obese, first-time pregnancies, advanced maternal age, alcohol consumption and smoking, low physical activity, or a genetic predisposition [4,5,6].

Recent evidence suggests that environmental factors, such as exposure to ambient air pollution and environmental chemicals, may also contribute to hypertensive disorders. Among these chemicals, phthalates—a group of non-persistent chemicals commonly used in consumer and personal care products—were linked to widespread exposure in pregnant women [7,8]. Phthalates, ubiquitously utilized in daily life to augment the durability of plastics, are found in an array of products: vinyl flooring, lubricating oils, plastic packaging, building materials, medical devices, and personal care items such as soaps, cosmetics, shampoos, and hair products. Notwithstanding their practical benefits, phthalates are acknowledged to be endocrine-disrupting compounds, and their exposure has been linked to elevated blood pressure and an amplified risk of cardiovascular diseases [9,10]. Exposure to phthalates can occur through multiple pathways: inhalation (including air, dust, and fragrance), ingestion (encompassing dietary ingestion and incidental ingestion), and dermal absorption (incorporating air-to-skin transport, use of personal care products, and contact with contaminated surfaces) [7,11,12]. Given their potential adverse effects on human health, certain phthalates are prohibited in some jurisdictions. 

Previous studies examining the association between phthalate metabolites and gestational BP and pregnancy-induced hypertension (PIH) have yielded mixed results. In an Ohio-based birth cohort study, prenatal urinary monobenzyl phthalate (MBzP) was associated with increased diastolic BP and the risk of PIH diseases [13]. However, two European studies did not replicate these findings [14,15]. Warembourg et al. (2019) observed negative correlations between monoethyl phthalate (MEP) and mono-iso-butyl phthalate (MiBP) concentrations and systolic BP [15]. In contrast, Philips et al. (2019) did not identify any consistent associations between early-pregnancy phthalate metabolite levels and BP or gestational hypertensive disorders [14]. Additionally, two Chinese studies with distinct background exposure levels also reported inconsistent results. Gao et al. (2021) found that exposure to specific phthalate metabolites or diesters during the first trimester raised BP in the third trimester [16], whereas Han et al. (2019) observed no significant associations between phthalate exposure and BP during pregnancy for the general population [17]. However, they noted that among pregnant women carrying male fetuses, first-trimester MiBP exposure was linked to increased second-trimester diastolic BP. 

While some epidemiological studies have reported associations between gestational urinary phthalate concentrations and elevated BP or increased HDP risk, others have not. To better understand the reasons behind these divergent findings, further investigation is necessary to determine the potential adverse effects of phthalates on pregnancy outcomes. As such, we conducted a systematic review and meta-analysis of human epidemiological studies to evaluate the relationship between gestational urinary phthalate levels, BP during pregnancy, and the risk of HDP. 

## 2. Materials and Methods

The present study adhered to PRISMA guidelines (http://www.prisma-statement.org/ (accessed on 5 March 2023)) (Supplementary Material, Table S1), and was not registered with PROSPERO in advance.

### 2.1. Search Strategy

We conducted a search for studies published up to 5 March 2023 using three databases: Embase, PubMed, and Web of Science. Relevant keywords were determined based on the PECO framework [18]. To generate search terms, we combined keywords that represented exposure and outcome components within the PECO framework. The following search terms were employed: (gestational phthalate exposure OR phthalates OR urinary phthalate metabolites OR maternal urinary phthalate concentrations) AND (blood pressure OR cardiometabolic indices OR hypertensive diseases of pregnancy OR pregnancy-induced hypertension OR maternal hemodynamics OR gestational hypertensive disorders OR gestational hypertension OR preeclampsia). These terms were used to identify English-language original articles reporting the effects of phthalate exposure on BP during pregnancy or risk of HDP. A comprehensive list of search terms utilized across various databases can be found in Supplementary Materials, Table S2.

No restrictions were placed on study designs during the search process. Relevant studies were also identified by screening reviews and reference lists of search results. After removing duplicates, titles and abstracts underwent an initial screening, followed by a full-text review. Two authors (MYZ and JCQ) independently assessed all articles, with their findings reaching a consensus. In cases of disagreement, a third reviewer (CYH) examined the article and made the final decision.

### 2.2. Inclusion and Exclusion Criterion

In alignment with the PECO elements, we applied the following inclusion criteria: (1) the population (P) consisted of pregnant women; (2) exposure (E) was determined by phthalate exposure measured from biological samples; (3) the comparator (C) involved a group with lower exposure levels; (4) the outcomes (O) included BP during pregnancy and risk of HDP, which were verified by physicians, medical records, or according to the International Classification of Diseases codes; and (5) the study was accessible as a full text and in English. The exclusion criteria included: (1) studies focusing on the general population; (2) studies concentrating on occupational exposure; and (3) studies without a reference group. 

### 2.3. Data Extraction and Quality Assessment

Two authors (MYZ and JCQ) independently carried out the data extraction and quality assessment. From each eligible study, we collected the source (author name and publication year), location, study period, study design, phthalates examined, exposure assessment, exposure period, outcomes, covariates adjusted for, and main findings. Additionally, we contacted several corresponding study authors to request supplementary results not included in their published articles but relevant to our research. Additionally, if necessary, we contacted several corresponding study authors to request supplementary results not included in their published articles but relevant to our research.

In our systematic review and meta-analysis, we assessed the quality of the incorporated studies by employing the Newcastle–Ottawa Scale (NOS) specifically designed for observational research [19]. The NOS contains eight elements, organized into three distinct categories: (I) selection of study groups; (II) group comparability; and (III) interest-specific exposure or outcomes. Individual categories receive a score, with the highest possible being four, two, and three stars, adding up to a maximum of nine stars. Utilizing this scale, we categorized studies as high-quality (≥7 stars), moderate (4–6 stars), or low-quality (≤3 stars) [20]. Any differences in determining the quality of the studies were settled through achieving a consensus.

### 2.4. Statistical Analysis 

We carried out a meta-analysis employing a random-effects model to accommodate variations both within and across studies. By utilizing the reported odds ratios (ORs) and their associated 95% confidence intervals (95% CIs), we calculated the combined ORs for the binary outcome of HDP. Additionally, coefficients from linear regressions were combined in a meta-analysis to estimate the effects of phthalate exposure on continuous outcomes, including diastolic and systolic BP. We performed separate meta-analyses for each trimester-specific exposure (phthalate metabolite exposure during the first, second, and second trimesters, or the full pregnancy mean) and outcomes (diastolic BP and systolic BP) when at least three studies were available. Given the limited number of studies addressing various HDP types, we included all research on HDP (e.g., GH and PE as distinct outcomes or a mix of these conditions), regardless of the employed definition, in order to provide an adequate basis for random-effects meta-analyses. 

For the purpose of facilitating effect estimate comparisons across studies, we transformed the documented ORs and 95% CIs to align with a standardized exposure increment (per natural log-unit increase). We assessed statistical heterogeneity among the selected studies using the Q-test and the I^2^ statistic (range: 0–100%). I^2^ values were classified as low, medium, or high based on values of 25, 50, or 75%, respectively [21]. We employed funnel plot asymmetry and Egger’s test to evaluate publication bias. If fewer than five studies were included, publication bias was not assessed. We adopted a *p*-value of <0.05 for Egger’s test. If the *p*-value was less than 0.05, publication bias was present, and vice versa [22]. For analyses with >3 studies, sensitivity analyses were conducted by systematically excluding one study at a time and recalculating summary effect sizes. All analyses were performed using Stata software version 16.1 (StataCorp, Texas, USA). All *p*-values were two-sided with a significance level set at 0.05.

## 3. Results

**Figure 1:**
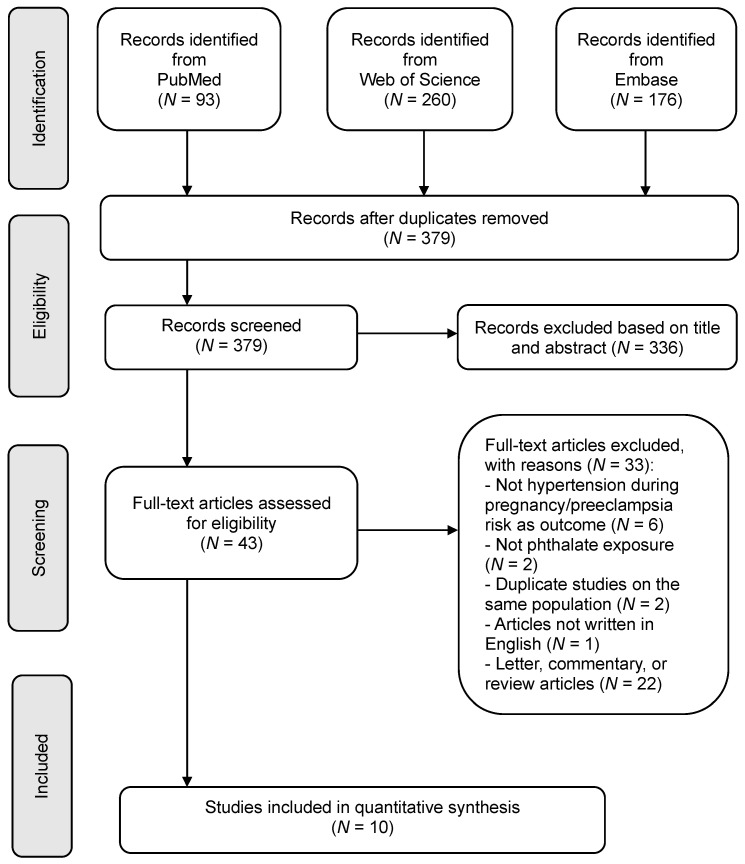
Flow diagram of systematic literature search through 31 March 2023.

The PRISMA flowchart, illustrating our literature search process, can be found in Figure 1. Our study encompassed a total of 379 studies, incorporating the outcomes of keyword searches. Following an in-depth examination of the full texts, 43 articles were deemed potentially suitable for inclusion in the meta-analysis. Finally, the present study included a total of 10 studies for the meta-analysis. Of the 10 included studies, 6 were performed in North America [9,13,14,23,24,25], 2 were from Europe [15,26], and the remaining 2 were from Asia [16,17]. The studies incorporated in our analysis were published between 2015 and 2021, with participant numbers ranging from 152 to 3273. Among these, nine were cohort studies, while one was a cross-sectional study. Each study assessed phthalate exposure using maternal urinary samples as the measurement method. 

### 3.1. Study Characteristics 

The majority (N = 7) of the included studies documented preeclampsia [9,13,14,16,23,24,26]. Additionally, four studies [13,23,24,26] reported on PIH, five studies [13,14,23,24,26] reported on GH, three studies [15,17,25] reported on blood pressure during pregnancy, five studies [13,16,23,24,26] reported on eclampsia, while only one study presented results for HELLP syndrome [13]. Three studies gathered multiple (three) urine samples during the gestational period [9,16,17]. Two studies gathered two urine samples during the second and second trimesters [15,25] and the first and second trimesters [23], respectively. Four studies gathered single urine sample during the first trimester [14] and second trimester [13,24,26], respectively. Table 1 provides the primary information for the eligible studies. 

The Newcastle–Ottawa Scale (NOS), ranging from 0 to 9 stars, was utilized to evaluate the quality of the studies, with a higher number of stars indicating superior quality. As shown in Table 2, all studies were assessed as high-quality (>7) using the NOS tool. 

### 3.2. Main Meta-Analysis Findings

The meta-analysis synthesized outcomes regarding the associations between multiple different phthalates, BP during pregnancy, and HDP risk, which are presented in Table 3 below.

#### 3.2.1. Phthalates Exposure during Pregnancy and BP

The aggregated findings on the impact of trimester-specific phthalates exposure throughout pregnancy on BP displayed varying outcomes. Some demonstrated a negative association with both diastolic and systolic BP. The first trimester MBP exposure was positively associated with the mean systolic BP and diastolic BP of the second and the second trimester (β = 1.05, 95% CI: 0.27, 1.83, I^2^ = 93%; β = 0.40, 95% CI: 0.05, 0.74, I^2^ = 71%), respectively. The second trimester MBzP exposure was positively associated with the systolic BP and diastolic BP of the second trimester (β = 0.57, 95% CI: 0.01, 1.13, I^2^ = 0; β = 0.70, 95% CI: 0.27, 1.13, I^2^ = 0), respectively. In contrast, the first trimester MEHP exposure was negatively associated with the mean systolic BP and diastolic BP of the second and the second trimester (β = −0.32, 95% CI: −0.60, −0.05, I^2^ = 0; β = −0.32, 95% CI: −0.60, −0.05, I^2^ = 0), respectively.

#### 3.2.2. Phthalates Exposure during Pregnancy and Risk of HDP

The collective findings indicate that trimester-specific phthalate exposure during pregnancy was not associated with HDP, with the exception of monoethyl phthalate (MEP) exposure. The combined effect estimates ranged from 1.01 to 1.18, with a low 95% confidence interval (CI) spanning from 0.86 to 1.02 and a high 95% CI extending from 1.09 to 1.41. However, almost all these combined effect estimates were not statistically significant. The only exception was MEP exposure, which was found to be associated with an increased risk of HDP (OR = 1.12, 95% CI = 1.02 to 1.23, I^2^ = 26%). 

### 3.3. Sensitivity Analysis and Publication Bias

The sensitivity analysis results demonstrated that the combined effect estimates remained generally stable when individual studies were excluded. Due to the limited number of studies included for each exposure and outcome combination, funnel plots were not conducted. Nonetheless, for all exposures, the *p*-values from Egger’s tests were not statistically significant (*p* > 0.05), suggesting no evidence of publication bias. However, given the relatively small number of studies in our meta-analysis, these test results should be interpreted cautiously, as they may lack the power to detect publication bias in meta-analyses with a limited number of studies [27].

## 4. Discussion

In this meta-analysis, we synthesized data from 10 human epidemiological studies to investigate the connection between phthalate exposure, BP, and HDP risk. Our findings suggest that, aside from MBP, MBzP, and MEHP, the majority of phthalate metabolites show no association with BP. Notably, only MEP exposure was associated with a heightened risk of HDP.

The impact of phthalate exposure on BP during pregnancy and HDP risk has attracted considerable interest in recent years. Soomro et al. found that prenatal exposure to phthalates, particularly MBP and MEP, could play a vital role in PIH [26]. Werner et al. proposed that early mid-pregnancy exposure to MBzP correlated with increased diastolic BP and HDP [13]. Warembourg et al. (2019) reported an association between phthalate metabolite exposure and decreased systolic and diastolic BP during pregnancy, particularly in the second trimester [15]. This observation is consistent with results showing an association between MEHP exposure and reduced systolic and diastolic BP. Although evidence on gestational BP changes related to phthalate exposure is somewhat conflicting, there is consistent support from prior studies involving children and nonpregnant adults that DEHP [28,29], MBP (a metabolite of DBP) [23,28], and MBzP [30,31] correlate with BP changes.

Numerous factors could account for the discrepancies observed in the results of prior studies. Inconsistencies between studies investigating the same phthalate might arise from inadequate statistical power, variation in outcome assessment, divergent analysis methods, or differing study populations. While the majority of studies assessed phthalate levels during one trimester, the timing of exposure evaluation was not consistent across studies. It is well-established that phthalate plasma/serum concentrations decrease as pregnancy progresses due to the expansion of plasma volume and the distribution of phthalates into the fetal compartment [13].

In terms of outcomes, three studies exclusively examined BP during pregnancy, one study focused on PE, and others investigated GH or other HDPs. Only a single study explored the association with repeated BP measurements throughout pregnancy [17]. Recently, women participating in the Programming Research in Obesity, Growth, Environment, and Social Stressors (PROGRESS) study were followed for up to 72 months postpartum to assess the associations between gestational phthalate exposure and postpartum outcomes. Wu et al. (2021) found that combined phthalate mixture levels were positively associated with elevated postpartum BP up to 72 months postpartum. This finding highlights the significance of examining women’s health beyond pregnancy when evaluating the potential effects of phthalate exposure [25]. 

The precise mechanisms underlying phthalate-induced elevated BP remain uncertain; however, current research provides some understanding. Phthalate metabolites function as agonists for PPARγ (peroxisome proliferator-activated receptor gamma), known to inhibit the renin-angiotensin-aldosterone system, an essential BP regulator [32,33]. Phthalate exposure has been associated with heightened oxidative stress in pregnant women [34], potentially affecting the release of circulating angiogenic factors. These factors not only serve as predictors for PE [35,36] but are also linked to PIH [37]. Furthermore, phthalates may impact BP by modifying thyroid hormone levels during pregnancy. A previous study found a connection between urinary phthalates and decreased serum thyroxine in pregnant women [38], which has been demonstrated to raise the risk of PE [39]. Additionally, phthalate-triggered inflammatory responses might play a significant role in GH. An earlier study among pregnant women uncovered a relationship between specific urinary phthalate metabolites and increased levels of inflammatory cytokines [40]. This heightened production of inflammatory cytokines could have a crucial role in GH or PE [41].

### 4.1. Strengths and Limitations

Meta-analyses can offer more accurate conclusions than individual studies by increasing statistical power, which is particularly crucial for rare outcomes. In our meta-analysis, we identified several significant relationships between phthalates and BP during pregnancy and risk of HDP, even though many individual studies failed to do so. The robustness of our findings was evident as the exclusion of individual studies typically had minimal impact on the results. Additionally, our meta-analysis showed no significant signs of small-study bias.

However, our study has several limitations that should be considered when interpreting the results. First, while we included all available studies in three databases examining the effect of phthalates on BP during pregnancy and the risk of HDP up to 2023, insufficient data on this research topic prevented us from conducting subgroup analyses based on study design, location, and offspring gender. Second, the studies were conducted in various geographic regions and populations, leading to differences in phthalate exposure. This factor may further affect the measured concentrations of these phthalates at different times. Furthermore, the range of exposure varied among the included studies, which could influence the meta-analysis results. Third, we exclusively included studies that assessed phthalate levels in urine samples to maintain consistency and comparability in our analysis. Although phthalate metabolites in urine are considered suitable biomarkers for short-term exposure to parent compounds due to their rapid excretion and short half-life in humans [42], urinary measurements may not accurately reflect long-term exposure. Lastly, some studies combined GH and PE, preventing us from including these studies in separate meta-analyses for HDP as a distinct outcome. 

### 4.2. Recommendations for Future Study

While some epidemiological evidence and potential mechanisms suggest that phthalates may be risk factors for elevated BP during pregnancy and HDP, additional research is necessary to verify this relationship and guide medical recommendations and environmental policies. Such research faces challenges, including logistical issues related to sample collection, timing, and the selection of specific phthalate metabolites for investigation. Acquiring well-timed samples from pregnant women and confirming their HDP diagnosis is both difficult and costly. Prior studies have indicated variations or interactions influenced by factors such as parity and fetal sex [13,43]. To develop a comprehensive understanding of HDP risk, these factors, among others, should be considered. Future research should, at a minimum, assess the modifying effects of fetal sex, parity, and race/ethnicity, and include the timing of phthalate measurements during pregnancy as a covariate in analyses. Accounting for essential confounders, such as maternal age, smoking status, and pre-pregnancy BMI, is crucial, as is exploring the modifying influence of diet and physical activity [44,45]. Investigating the relationship between phthalates and HDP in diverse racial and ethnic populations is essential to evaluate risk and develop targeted prevention strategies.

To fully understand the effects of phthalates on health outcomes, future research must take into account their cumulative burden. Methods for exposure assessment and strategies for categorizing phthalates during risk assessment are being developed [46,47]. As epidemiological studies increasingly measure multiple phthalate metabolites, developing methods for modeling exposure mixtures will become increasingly important. Available statistical methods include, but are not limited to, BKMR [48], environmental risk score [49], supervised principal component analysis followed by classification and regression tree [50], toxicant score [51], joint WQS regression [52,53], and machine learning methods such as lasso or adaptive elastic net [50]. When selecting the appropriate method, the correlation structure between phthalates is crucial. For instance, joint WQS displays strong sensitivity and specificity for identifying predictors within a correlated mixture, while other methods might be more suitable for non-correlated or weakly correlated exposures [54,55]. 

## 5. Conclusions

Our meta-analysis determined that there was a strong association between several phthalate metabolites (MBP, MBzP, and MEHP) and BP during pregnancy, while MEP was found to be associated with an increased risk of HDP. However, these results should be interpreted cautiously due to the relatively small number of included studies. Given the considerable maternal and fetal morbidity and mortality associated with HDP, this study has significant public health implications. Nonetheless, additional research is required to clarify the underlying mechanisms.

## Figures and Tables

**Table 1:** Characteristics of the included studies on the associations of phthalate exposure with blood pressure and risk of hypertension during pregnancy.

Source	Location	Study Period	Study Design	Sample Size	Phthalates Examined	Exposure Assessment	Exposure Period	Outcome(s)	Covariates Adjusted for	Main Findings
[9]	U.S.	2011	Cohort	50	MEHP; MEHHP; MEOHP; MECPP; MBzP; MBP; MiBP; MEP; MCPP; ΣDEHP	Maternal urine sample	1st, 2nd and 3rd trimesters	PE	Age, race/ethnicity, prepregnancy BMI, health insurance category, education, smoking status during pregnancy, parity, gestational diabetes, prior history of PE	Several phthalate metabolites were significantly associated with increased risk of PE
[13]	U.S.	2003–2006	Cohort	369	MBzP; MMP; MEP; MiBP; MnBP; MEHP; MEOHP; MEHHP; MECPP; MBP; MCPP; ΣDEHP	Maternal urine sample	2nd trimester	PIH (GH; PE; eclampsia; HELLP syndrome)	Race, age, house hold income, education, marital status, serum cotinine concentrations, parity, BMI at 16 weeks gestation, self-reported use of medications for high BP	Maternal urinary MBzP concentrations may be associated with increased diastolic BP and risk of pregnancy-induced hypertensive diseases
[14]	U.S.	2004–2005	Cohort	1396	Phthalic acid/LMW/HMW/DEHP/DNOP metabolite	Maternal urine sample	1st trimester	Gestational hypertensive disorders (GH; PE)	Maternal age, maternal pre-pregnancy BMI, parity, ethnicity, education, maternal smoking, maternal alcohol, folic acid supplementation, gestational age at time of measurement and creatinine	Phthalate metabolite concentrations were not associated with gestational hypertensive disorders
[15]	Spain	2014–2015	Cross-sectional	152	MEP; MiBP; MnBP; MBzP; MEHP; MEHHP; MEOHP; MECPP; ΣDEHP; OH-MiNP; OXO-MiNP	Maternal urine sample	2nd and 3rd trimesters	BP during pregnancy	Study center, age, ethnicity, marital status, pre-pregnancy BMI, BMI at examination, maternal height, education, working status, parity, physical activity, fruits and vegetables consumption, ultra-processed food consumption, gestational age, smoking status at examination	
[16]	China	-	Cohort	3273	DMP; DEP; DBP; MEHP; MEOHP; MBP; MMP; MEP; MBzP; MEHHP; BBzP; ∑DEHP	Maternal urine sample	1st, 2nd and 3rd trimesters	HDOP (GH; PE; severe PE; eclampsia)	Age, pre-pregnancy BMI, education, residence, race, occupation type, monthly household income, drinking during pregnancy, smoking during pregnancy, primiparous	Exposure to a single phthalate metabolite or a specific diester during the first trimester of pregnancy elevated BP in the third trimester. However, inverse relationships were revealed for some phthalate metabolites, which were inconsistent with the results of their diesters
[17]	China	2013–2015	Cohort	636	MiBP; ΣDEHP; MMP; MEP; MnBP; MBzP; MEHP; MEOHP; MEHHP; MECPP; ΣLMW	Maternal urine sample	1st, 2nd and 3rd trimesters	BP during pregnancy	Pre-pregnancy BMI, household income, GDM, fetus gender, age, parity	Exposure to phthalates was positively related to BP in pregnant women
[23]	U.S.	2010–2012	Cohort	668	MEP; MCPP; MiBP MBP; ∑DEHP; MBzP; MEHP; ∑DEHTP	Maternal urine sample	1st and 3rd trimesters	PIH, GH; PE; eclampsia	Study center, race, age at delivery, household income, highest level of education, marital status, cigarette smoking in the first trimester, pre-pregnancy BMI, and parity	Several phthalate metabolite concentrations were significantly associated with PIH and greater increases in systolic BP across pregnancy
[24]	U.S.	2003–2006	Cohort	388	MEP; MBP; MiBP; MBzP; MCPP; ∑DEHP	Maternal urine sample	2nd trimester	PIH, GH; PE; eclampsia	Age, race/ethnicity, annual household income, smoking status, marijuana use, pre-pregnancy BMI, parity, gestational week	No relationship was noted between any EDC compound and PIH disorders
[25]	Mexico	2007–2011	Cohort	892	∑DEHP; MECPP; MBzP; ∑DBP; MCPP; MEP; MCNP; ∑DiNP; ∑DiBP	Maternal urine sample	2nd and 3rd trimesters	BP during pregnancy	Age, SES, education, parity, second trimester BMI, second trimester height, second trimester BP, seasonality, and gestation age	Exposure to phthalates and phthalate biomarkers was associated with higher BP during late pregnancy
[26]	France	2003–2006	Cohort	604	MEP; MBP; MiBP; MECPP; MEHHP; MEOHP; MEHP; MBzP; MCOP; MCPP; MCNP; ΣDEHP	Maternal urine sample	2nd trimester	PIH, GH; PE; eclampsia	Smoking, age, BMI, education level, gestational age, number of siblings	Prenatal exposure to some phthalates, including MEP and MBP, might play a role in pregnancy induced hypertension

Abbreviations: U.S., United States of America; MEP, Monoethyl phthalate; MBP, Mono-n butyl phthalate; MiBP, Mono-isobutyl phthalate; MECPP, Mono (2-ethyl-5-carboxypentyl) phthalate; MEHHP, Mono (2-ethyl-5-hydroxyhexyl) phthalate; MEOHP, Mono (2-ethyl-5-oxohexyl) phthalate; MEHP, Mono (2-ethylhexyl) phthalate; MBzP, Monobenzyl phthalate; MCOP, Monocarboxy-isooctyl phthalate; MCPP, Mono (3-carboxypropyl) phthalate; MCNP, Monocarboxy-isononyl phthalate; DEHP, di(2-ethylhexyl) phthalate (∑DEHP = MECPP, MEHP, MEHHP, and MEOHP); DEHTP, di-(2-ethylhexyl) terephthalate; DMP, dimethyl phthalate; DEP, diethyl phthalate; DBP, dibutyl phthalate; BBzP, butyl benzyl phthalate; MMP, monomethyl phthalate; DiNP, diisononyl phthalate (ΣDiNP = MONP + MCOP); DiBP, diisobutyl phthalate (ΣDiBP = MHiBP + MiBP); DBP, dibutyl phthalate; ΣLMW: the molar sum of MEP; MnBP, mono-n-butyl phthalate; DNOP, di-n-octylphthalate; HMW, high molecular weight; HMW, high molecular weight; OH-MiNP, Mono-4-methyl-7-hydroxyoctyl phthalate; OXO-MiNP, Mono-4-methyl-7-oxooctyl phthalate; PIH, pregnancy induced hypertension syndrome; GH, gestational hypertension; PE, pre-eclampsia; BMI, body mass index; EDC, endocrine disrupting chemical; BP, blood pressure.

**Table 2:** Quality assessment of included studies by the Newcastle–Ottawa scale.

Study Design	Reference	Selection				Comparability	Outcome/Exposure			Quality Score
		Item 1	Item 2	Item 3	Item 4	Item 5	Item 6	Item 7	Item 8	
Case-control	[9]	★	★		★	★★	★	★	★	8/9
Cohort	[13]	★	★	★	★	★	★	★		7/9
	[14]	★	★	★	★	★★	★	★		8/9
	[15]	★	★	★	★	★★	★	★	★	9/9
	[16]	★	★	★	★	★	★	★	★	8/9
	[17]	★	★	★	★	★	★	★		7/9
	[23]	★	★	★	★	★★	★	★		8/9
	[24]	★	★	★	★	★★	★	★		8/9
	[25]	★	★	★	★	★★	★	★		8/9
	[26]	★	★	★	★	★★	★	★		8/9

Notes: The NOS has eight items grouped in three domains: (I) selection of study groups; (II) comparability of the groups; and (III) exposure or outcomes of interest. Each domain is scored with a maximum of four, two, and three stars, respectively; thus, the total score for the scale sums up to nine stars. Based on this scale, the studies were classified as high quality (≥7 stars), moderate (4–6 stars), and low quality (≤3 stars).

**Table 3:** Meta-analytical summary estimates of associations between phthalate exposure (each 1 ln unit increase) and blood pressure and risk of hypertension during pregnancy. (NOTE: the results were grouped by time of exposure and outcome measurement and outcome of interest).

Time of Outcome Measurement	Time of Exposure Measurement	Systolic Blood Pressure				Diastolic Blood Pressure				Hypertensive Disorders of Pregnancy			
		N	Pooled Estimates (β)	I^2^ (%)	Tau^2^	N	Pooled Estimates (β)	I^2^ (%)	Tau^2^	N	Pooled Estimates(OR)	I^2^ (%)	Tau^2^
MBP													
T1	T1	<3	NA			<3	NA			4	1.07 (0.95, 1.20)	0	0.0005
	T2 + T3	3	1.05 (0.27, 1.83) *	93	0.3780	3	0.40 (0.05, 0.74) *	71	0.0514	<3	NA		
T2	T2	<3	NA			<3	NA			<3	NA		
Average		<3	NA			<3	NA			5	1.04 (0.92, 1.17)	0	0.0018
MBzP													
T1	T1	<3	NA			<3	NA			4	1.01 (0.94, 1.09)	0	0.0009
	T2 + T3	3	0.10 (−0.40, 0.59)	32	0.1375	3	0.06 (−0.28, 0.40)	41	0.0625	<3	NA		
T2	T2	<3	NA			<3	NA			<3	NA		
	T3	4	0.57 (0.01, 1.13) *	0	0.0107	4	0.70 (0.27, 1.13) *	0	0.0012	<3	NA		
Average		<3	NA			<3	NA			6	1.09 (0.99, 1.20)	8	0.0109
MCPP													
T1	T1	<3	NA			<3	NA			3	1.18 (0.99, 1.41)	18	0.0117
T2	T3	3	0.63 (–1.14, 2.40)	72	1.7700	3	0.18 (−0.51, 0.86)	0	0.0042	<3	NA		
Average		<3	NA			<3	NA			5	1.02 (0.86, 1.22)	0	0.0066
MEHP													
T1	T2 + T3	3	−0.32 (−0.60, −0.05) *	0	0.0163	3	−0.38 (−0.61, −0.15)	32	0.0139	<3	NA		
MEHHP													
T1	T1	<3	NA			<3	NA			3	1.02 (0.89, 1.17)	0	0.0002
Average		<3	NA			<3	NA			3	1.10 (0.96, 1.26)	0	<0.0001
MEP													
T1	T1	<3	NA			<3	NA			4	1.12 (1.02, 1.23)	26	0.0104
	T2 + T3	3	0.14 (−0.14, 0.41)	34	0.0200	3	0.06 (−0.20, 0.32)	48	0.0292	<3	NA		
T2	T2	3	−0.12 (−0.95, 0.72)	44	0.3378	3	−0.16 (−0.78, 0.45)	36	0.1702	<3	NA		
	T3	5	0.21 (−0.61, 1.04)	50	0.6418	5	0.22 (−0.27, 0.71)	35	0.1897	<3	NA		
Average		<3	NA			<3	NA			6	1.06 (0.97, 1.15)	0	0.0055
MIBP													
T1	T1	<3	NA			<3	NA			3	1.14 (0.94, 1.39)	32	0.0292
T2	T3	3	0.04 (−0.91, 0.98)	26	0.2691	3	0.17 (−0.38, 0.71)	0	0.0652	<3	NA		
Average		<3	NA			<3	NA			4	1.11 (0.93, 1.33)	48	0.0606
MMP													
T1	T1	<3	NA			<3	NA			3	1.05 (0.93, 1.19)	0	0.0004
Average		<3	NA			<3	NA			3	1.02 (0.92, 1.14)	0	0.0009
ΣDEHP													
T1	T2 + T3	3	0.27 (−0.11, 0.66)	0	0.0229	3	−0.01 (−0.30, 0.28)	0	0.0088	<3	NA		
T2	T2	3	−0.02 (−0.58, 0.53)	0	0.0041	3	−0.22 (−0.63, 0.19)	0	0.0007	<3	NA		
	T3	5	0.25 (−0.39, 0.90)	15	0.1802	5	−0.13 (−0.73, 0.47)	22	0.2403	<3	NA		
Average		<3	NA			<3	NA			4	1.11 (0.96,1.28)	0	0.0122

* Indicates statistically significant association. Abbreviations: MBP, Mono-n butyl phthalate; MBzP, Monobenzyl phthalate; MCPP, Mono (3-carboxypropyl) phthalate; MEHP, Mono (2-ethylhexyl) phthalate; MEHHP, Mono (2-ethyl-5-hydroxyhexyl) phthalate; MEP, Monoethyl phthalate; MiBP, Mono-isobutyl phthalate; MMP, monomethyl phthalate; DEHP, di(2-ethylhexyl) phthalate (∑DEHP = MECPP, MEHP, MEHHP, and MEOHP); T1, first trimester; T2, second trimester; T3, third trimester.

## Supplementary Materials

The following supporting information can be downloaded at: https://www.mdpi.com/article/10.3390/metabo13070812/s1, Table S1. Literature search strategy (dated to 5 March 2023); Table S2. Preferred Reporting Items for Systematic reviews and Meta-Analysis (PRISMA) 2009 Checklist.
