# Supplementary material for: The Association between Maternal Urinary Phthalate Concentrations and Blood Pressure in Pregnancy: A Systematic Review and Meta-Analysis
Source: Metabolites. 2023 Jun 30;13(7):812. doi: 10.3390/metabo13070812 (PMC10384991; doi:10.3390/metabo13070812)
Supplement: Supplementary file 1 [file metabolites-13-00812-s001.zip › metabolites-2428819-supplementary.pdf]

SUPPLEMENTAL MATERIAL

"The association between maternal urinary phthalate concentrations and blood pressure in pregnancy: A systematic review and meta-analysis"

Mengyue Zhang, Jianchao Qiao, Pinpeng Xie, Zhuoyan Li, Chengyang Hu, Fei Li

TABLE OF CONTENTS

**Table S1.** Literature search strategy (dated to March 5, 2023)
**Table S2.** Preferred Reporting Items for Systematic reviews and Meta-Analysis (PRISMA) 2009 Checklist

Table S1. Literature search strategy (dated to March 10, 2020).

Database	Search strategies	**Results**
PubMed	(((((((((((((("Hypertension, Pregnancy-Induced"[Mesh]) OR (Hypertension, Pregnancy-Induced[Title/Abstract])) OR (Hypertension, Pregnancy Induced[Title/Abstract])) OR (Pregnancy-Induced Hypertension[Title/Abstract])) OR (Pregnancy Induced Hypertension[Title/Abstract])) OR (Hypertensions, Pregnancy Induced[Title/Abstract])) OR (Induced Hypertension, Pregnancy[Title/Abstract])) OR (Induced Hypertensions, Pregnancy[Title/Abstract])) OR (Gestational Hypertension[Title/Abstract])) OR (Hypertension, Gestational[Title/Abstract])) OR (Transient Hypertension, Pregnancy[Title/Abstract])) OR (Hypertension, Pregnancy Transient[Title/Abstract])) OR (Pregnancy Transient Hypertension[Title/Abstract])) OR ((((((((((((((((((((((((((((((((("Pre-Eclampsia"[Mesh]) OR (Pre-Eclampsia[Title/Abstract])) OR (Pre Eclampsia[Title/Abstract])) OR (Preeclampsia[Title/Abstract])) OR (Pregnancy Toxemias[Title/Abstract])) OR (Pregnancy Toxemia[Title/Abstract])) OR (Toxemia, Pregnancy[Title/Abstract])) OR (Edema-Proteinuria-Hypertension Gestosis[Title/Abstract])) OR (Edema Proteinuria Hypertension Gestosis[Title/Abstract])) OR (Gestosis, Edema-Proteinuria-Hypertension[Title/Abstract])) OR (Hypertension-Edema-Proteinuria Gestosis[Title/Abstract])) OR (Gestosis, Hypertension-Edema-Proteinuria[Title/Abstract])) OR (Hypertension Edema Proteinuria Gestosis[Title/Abstract])) OR (Toxemia Of Pregnancy[Title/Abstract])) OR (Pregnancies, Toxemia[Title/Abstract])) OR (Of Pregnancy, Toxemia[Title/Abstract])) OR (Pregnancies, Toxemia Of[Title/Abstract])) OR (Pregnancy, Toxemia Of[Title/Abstract])) OR (Toxemia Of Pregnancies[Title/Abstract])) OR (EPH Complex[Title/Abstract])) OR (EPH	93

	Toxemias[Title/Abstract])) OR (EPH Toxemia[Title/Abstract])) OR (Toxemia, EPH[Title/Abstract])) OR (Toxemias, EPH[Title/Abstract])) OR (EPH Gestosis[Title/Abstract])) OR (Gestosis, EPH[Title/Abstract])) OR (Toxemias, Pregnancy[Title/Abstract])) OR (Preeclampsia Eclampsia 1[Title/Abstract])) OR (1, Preeclampsia Eclampsia[Title/Abstract])) OR (1s, Preeclampsia Eclampsia[Title/Abstract])) OR (Eclampsia 1, Preeclampsia[Title/Abstract])) OR (Eclampsia 1s, Preeclampsia[Title/Abstract])) OR (Preeclampsia Eclampsia 1s[Title/Abstract]))) AND (((((((((("Diethylhexyl Phthalate"[Mesh]) OR ("Dibutyl Phthalate"[Mesh])) OR (Diethylhexyl Phthalate[Title/Abstract])) OR (Dibutyl Phthalate[Title/Abstract])) OR (phthalate*[Title/Abstract])) OR (phthalic*[Title/Abstract])) OR (endocrine disruptor*[Title/Abstract])) OR (endocrine disrupting chemical*[Title/Abstract])) OR (PAE[Title/Abstract])) OR (PAEs[Title/Abstract]))	
Web of Science	TOPIC: (((Pregnancy-Induced Hypertension OR Pregnancy Induced Hypertension OR Gestational Hypertension OR Pregnancy Transient Hypertension) OR (Pre-Eclampsia OR Pre Eclampsia OR Preeclampsia OR Pregnancy Toxemias OR Pregnancy Toxemia OR Edema-Proteinuria-Hypertension Gestosis OR Edema Proteinuria Hypertension Gestosis OR Hypertension-Edema-Proteinuria Gestosis OR Hypertension Edema Proteinuria Gestosis OR Toxemia Of Pregnancy OR Toxemia Of Pregnancies OR EPH Complex OR EPH Toxemias OR EPH Toxemia OR EPH Gestosis OR Preeclampsia Eclampsia 1 OR Preeclampsia Eclampsia 1s)) AND (Diethylhexyl Phthalate OR Dibutyl Phthalate OR phthalate* OR phthalic* OR endocrine disruptor* OR endocrine disrupting chemical* OR PAE OR PAEs))	260
EMBASE	(('maternal hypertension'/exp OR 'maternal hypertension' OR 'Hypertension, Pregnancy-Induced':ab,ti OR 'Hypertension, Pregnancy Induced':ab,ti OR 'Pregnancy-Induced	176

	Hypertension':ab,ti OR 'Pregnancy Induced Hypertension':ab,ti OR 'Hypertensions, Pregnancy Induced':ab,ti OR 'Induced Hypertension, Pregnancy':ab,ti OR 'Induced Hypertensions, Pregnancy':ab,ti OR 'Gestational Hypertension':ab,ti OR 'Hypertension, Gestational':ab,ti OR 'Transient Hypertension, Pregnancy':ab,ti OR 'Hypertension, Pregnancy Transient':ab,ti OR 'Pregnancy Transient Hypertension':ab,ti) OR ('preeclampsia'/exp OR 'preeclampsia' OR 'Pre-Eclampsia':ab,ti OR 'Pre Eclampsia':ab,ti OR 'Preeclampsia':ab,ti OR 'Pregnancy Toxemias':ab,ti OR 'Pregnancy Toxemia':ab,ti OR 'Toxemia, Pregnancy':ab,ti OR 'Edema-Proteinuria-Hypertension Gestosis':ab,ti OR 'Edema Proteinuria Hypertension Gestosis':ab,ti OR 'Gestosis, Edema-Proteinuria-Hypertension':ab,ti OR 'Hypertension-Edema-Proteinuria Gestosis':ab,ti OR 'Gestosis, Hypertension-Edema-Proteinuria':ab,ti OR 'Hypertension Edema Proteinuria Gestosis':ab,ti OR 'Toxemia Of Pregnancy':ab,ti OR 'Pregnancies, Toxemia':ab,ti OR 'Of Pregnancy, Toxemia':ab,ti OR 'Pregnancies, Toxemia Of':ab,ti OR 'Pregnancy, Toxemia Of':ab,ti OR 'Toxemia Of Pregnancies':ab,ti OR 'EPH Complex':ab,ti OR 'EPH Toxemias':ab,ti OR 'EPH Toxemia':ab,ti OR 'Toxemia, EPH':ab,ti OR 'Toxemias, EPH':ab,ti OR 'EPH Gestosis':ab,ti OR 'Gestosis, EPH':ab,ti OR 'Toxemias, Pregnancy':ab,ti OR 'Preeclampsia Eclampsia 1':ab,ti OR '1, Preeclampsia Eclampsia':ab,ti OR '1s, Preeclampsia Eclampsia':ab,ti OR 'Eclampsia 1, Preeclampsia':ab,ti OR 'Eclampsia 1s, Preeclampsia':ab,ti OR 'Preeclampsia Eclampsia 1s':ab,ti)) AND ('phthalic acid'/exp OR 'phthalic acid' OR 'Diethylhexyl Phthalate':ab,ti OR 'Dibutyl Phthalate':ab,ti OR 'phthalate*':ab,ti OR 'phthalic*':ab,ti OR 'endocrine disruptor*':ab,ti OR 'endocrine disrupting chemical*':ab,ti OR 'PAE':ab,ti OR 'PAEs':ab,ti)	

Table S2. Preferred Reporting Items for Systematic reviews and Meta-Analysis (PRISMA) 2009 Checklist.

Section/topic	#	Checklist item	Reported on page #
**TITLE**			
Title	1	Identify the report as a systematic review, meta-analysis, or both.	1
**ABSTRACT**			
Structured summary	2	Provide a structured summary including, as applicable: background; objectives; data sources; study eligibility criteria, participants, and interventions; study appraisal and synthesis methods; results; limitations; conclusions and implications of key findings; systematic review registration number.	1
**INTRODUCTION**			
Rationale	3	Describe the rationale for the review in the context of what is already known.	2
Objectives	4	Provide an explicit statement of questions being addressed with reference to participants, interventions, comparisons, outcomes, and study design (PICOS).	2
**METHODS**			
Protocol and	5	Indicate if a review protocol exists, if and where it can be accessed (e.g., Web address), and, if available, provide registration information including registration number.	NA

registration			
Eligibility criteria	6	Specify study characteristics (e.g., PICOS, length of follow-up) and report characteristics (e.g., years considered, language, publication status) used as criteria for eligibility, giving rationale.	3
Information sources	7	Describe all information sources (e.g., databases with dates of coverage, contact with study authors to identify additional studies) in the search and date last searched.	3
Search	8	Present full electronic search strategy for at least one database, including any limits used, such that it could be repeated.	2
Study selection	9	State the process for selecting studies (i.e., screening, eligibility, included in systematic review, and, if applicable, included in the meta-analysis).	3
Data collection process	10	Describe method of data extraction from reports (e.g., piloted forms, independently, in duplicate) and any processes for obtaining and confirming data from investigators.	3
Data items	11	List and define all variables for which data were sought (e.g., PICOS, funding sources) and any assumptions and simplifications made.	3
Risk of bias in individual studies	12	Describe methods used for assessing risk of bias of individual studies (including specification of whether this was done at the study or outcome level), and how this information is to be used in any data synthesis.	3
Summary measures	13	State the principal summary measures (e.g., risk ratio, difference in means).	3

Synthesis of results	14	Describe the methods of handling data and combining results of studies, if done, including measures of consistency (e.g., I^2) for each meta-analysis.	3-4
Risk of bias across studies	15	Specify any assessment of risk of bias that may affect the cumulative evidence (e.g., publication bias, selective reporting within studies).	3
Additional analyses	16	Describe methods of additional analyses (e.g., sensitivity or subgroup analyses, meta-regression), if done, indicating which were pre-specified.	3-4
**RESULTS**			
Study selection	17	Give numbers of studies screened, assessed for eligibility, and included in the review, with reasons for exclusions at each stage, ideally with a flow diagram.	4-5
Study characteristi cs	18	For each study, present characteristics for which data were extracted (e.g., study size, PICOS, follow-up period) and provide the citations.	5-9
Risk of bias within studies	19	Present data on risk of bias of each study and, if available, any outcome level assessment (see item 12).	10
Results of individual studies	20	For all outcomes considered (benefits or harms), present, for each study: (a) simple summary data for each intervention group (b) effect estimates and confidence intervals, ideally with a forest plot.	9-10
Synthesis of	21	Present results of each meta-analysis done, including confidence intervals and measures of	9-12

results		consistency.	
Risk of bias across studies	22	Present results of any assessment of risk of bias across studies (see Item 15).	12
Additional analysis	23	Give results of additional analyses, if done (e.g., sensitivity or subgroup analyses, meta-regression [see Item 16]).	12
**DISCUSSION**			
Summary of evidence	24	Summarize the main findings including the strength of evidence for each main outcome; consider their relevance to key groups (e.g., healthcare providers, users, and policy makers).	12-14
Limitations	25	Discuss limitations at study and outcome level (e.g., risk of bias), and at review-level (e.g., incomplete retrieval of identified research, reporting bias).	13-14
Conclusions	26	Provide a general interpretation of the results in the context of other evidence, and implications for future research.	14-15
**FUNDING**			
Funding	27	Describe sources of funding for the systematic review and other support (e.g., supply of data); role of funders for the systematic review.	15
